# Maternal Serum Preconception Polychlorinated Biphenyl Concentrations and Infant Birth Weight

**DOI:** 10.1289/ehp.0901150

**Published:** 2009-10-22

**Authors:** Laurel E. Murphy, Audra L. Gollenberg, Germaine M. Buck Louis, Paul J. Kostyniak, Rajeshwari Sundaram

**Affiliations:** 1 *Eunice Kennedy Shriver* National Institute of Child Health and Human Development, Division of Epidemiology, Statistics and Prevention Research, Rockville, Maryland, USA; 2 State University of New York at Buffalo, Toxicology Research Center, Buffalo, New York, USA

**Keywords:** birth weight, developmental toxicant, early origins of disease, endocrine disruptors, polychlorinated biphenyls, preconception

## Abstract

**Background:**

Prenatal and postnatal polychlorinated biphenyl (PCBs) exposure has been associated with decrements in fetal and infant growth and development, although exposures during the preconception window have not been examined despite recent evidence suggesting that this window may correspond with the highest serum concentrations.

**Objectives:**

We assessed maternal serum PCB concentrations at two sensitive developmental windows in relation to birth weight.

**Methods:**

Serum samples were collected from 99 women as they began trying to become pregnant (preconception) and after a positive pregnancy test (prenatal); 52 (53%) women gave birth and represent the study cohort. Using daily diaries, women recorded sexual intercourse, menstruation, and home pregnancy test results until pregnant or up to 12 menstrual cycles with intercourse during the estimated fertile window. With gas chromatography with electron capture, 76 PCB congeners were quantified (nanograms per gram serum) and subsequently categorized by purported biologic activity. Serum PCBs were log-transformed and entered both as continuous and categorized exposures along with birth weight (grams) and covariates [smoking (yes/no), height (inches), and infant sex (male/female)] into linear regression.

**Results:**

A substantial reduction in birth weight (grams) was observed for women in the highest versus the lowest tertile of preconception antiestrogenic PCB concentration (β = −429.3 g, *p* = 0.038) even after adjusting for covariates (β = −470.8, *p* = 0.04).

**Conclusions:**

These data reflect the potential developmental toxicity of antiestrogenic PCBs, particularly during the sensitive preconception critical window among women with environmentally relevant chemical exposures, and underscore the importance of PCB congener–specific investigation.

Polychlorinated biphenyls (PCBs) are persistent lipophilic compounds that have bioaccumulated and biomagnified within the human food chain (Agency for Toxic Substances and Disease Registry 2000; Givens et al. 2007), resulting in ubiquitous exposure for many populations. Consumption of contaminated fish is a primary route of human exposure, and such consumption has been associated with adverse reproductive effects (e.g., time to pregnancy, menstruation) (Buck Louis et al. 2009; Mendola et al. 1997) and developmental effects (e.g., birth weight, birth defects, developmental delays) (Halldorsson et al. 2008; Jacobson et al. 1985; Mendola et al. 2005). A collective body of research relying on serum and plasma concentrations of PCBs in relation to reproductive and developmental outcomes corroborates earlier research relying on proxy exposures such as fish consumption, occupation, or residence. Specifically, prenatal PCBs have been associated with decrements in birth weight when measured in serum (Hertz-Picciotto et al. 2005; Sonneborn et al. 2008) or plasma (Halldorsson et al. 2008).

PCBs readily cross the placenta (Jacobson et al. 1984), underscoring the importance of the *in utero* or prenatal exposure for the developing embryo and fetus. Recognizing this sensitive window, investigators have attempted to quantify prenatal exposures by measuring PCBs in maternal blood obtained at some point during pregnancy (Hertz-Picciotto et al. 2005), after delivery (Sonneborn et al. 2008), or using cord blood to represent prenatal exposure (Tan et al. 2009). Other investigators have quantified PCBs in breast milk, given its high fat content coupled with the lipophilic nature of PCBs, as a measure of postnatal exposure for nursing infants (Patandin et al. 1998). Although the exact mechanism remains to be elucidated, PCBs are known to disrupt thyroid hormone signaling and other endocrine pathways critical to development that could lead to decreased *in utero* growth (Agency for Toxic Substances and Disease Registry 2000). In addition, growing evidence supports the manifestations of effects after exposures during earlier sensitive windows at later ages, such as at birth, during childhood, or in adulthood (Boisen et al. 2001; Skakkebaek et al. 2001). To a large extent, the timing of biospecimen collection for the quantification of PCB exposure has been ignored, given their assumed stability in blood during pregnancy. Recent evidence suggests that timing may be important, because concentrations decline from the preconception to postnatal sensitive windows (Bloom et al. 2007). If corroborated, effect sizes based on measurements later in pregnancy may be biased toward the null. To address this concern, we assessed the effect of PCB concentrations both before and shortly after conception in relation to birth weight and gestation. We further attempted to elucidate specific PCB congeners that may be associated with birth weight. To our knowledge, no previous studies have examined multiple PCB measurements during the pre- and periconception sensitive windows of development or by purported biologic activity of congener groupings in relation to birth weight and gestation.

## Materials and Methods

### Study population

A cohort of women planning pregnancies was recruited from a larger population-based cohort of anglers who previously participated in a study focusing on fish consumption and health (Vena et al. 1996). In 1995 and 1996, 2,637 participating women were recontacted and mailed introductory letters followed by telephone follow-up to ascertain their pregnancy intentions and willingness to participate in a study focusing on PCBs, time to pregnancy, and pregnancy outcomes; 1,031 (39%) women were successfully screened, of whom 244 (24%) were eligible for participation. Eligibility criteria included age 18–34 years, no physician-diagnosed infertility, and planning a pregnancy in the next 6 months. Among the 244 eligible women, 113 (46%) were recruited and enrolled into the cohort; another 14 were identified as already pregnant around enrollment and were excluded. Of the 99 remaining eligible women, 20 withdrew during the 12 months of attempting pregnancy. Of the 79 women completing the study follow-up, 69 became pregnant within the time frame of the study, and 55 (70%) of these pregnancies resulted in live births. Ten (13%) participants were unable to conceive after 12 menstrual cycles, and 14 (18%) experienced pregnancy losses. Human subject approval was awarded for the study protocol by the Institutional Review Board for the School of Medicine & Biomedical Sciences at the University at Buffalo, State University of New York, and individual written consent was obtained from all women before data collection.

### Data collection

Women completed baseline interviews on discontinuing contraception and completed daily diaries about menstruation, sexual intercourse, home pregnancy test results, and lifestyle behaviors (e.g., cigarette use and alcohol consumption). Research nurses instructed women in the use of home pregnancy kits designed to detect 50 mIU of human chorionic gonadotropin (hCG) in urine. As a part of the standardized protocol, women were instructed to test their urine for pregnancy on the day of expected menses, given that women’s menstrual cycles differ in length, and then again 1 week later regardless of the results of the first test. We estimated gestational length as the number of days between the estimated date of conception and delivery date. Specifically, conception was estimated to have occurred 14 days before the first positive pregnancy test. Birth weights were reported by mothers after delivery.

Women provided nonfasting blood samples (10 mL) for the quantification of PCBs at baseline (preconception) on completion of the standardized interview and enrollment into the cohort during 1996–1998, after a positive pregnancy test [prenatal: median, 6 weeks gestation; interquartile range (IQR), 2 weeks], and after delivery (postnatal: median, 6 weeks postdelivery; IQR, 2 weeks). Among the 55 women who became pregnant, serum samples in sufficient amounts for toxicologic analysis were available for 50 women during the preconception period, 52 women in the prenatal period, and 52 women in the postnatal period. Fifty-two (95%) children underwent a neurodevelopmental assessment at 2 years of age, and optional blood specimens were obtained for 19 (35%) children (Senn et al. 2005).

### Toxicologic analysis

Maternal serum samples were analyzed by one toxicologic laboratory for 76 congeners (64 single and 12 dieluting congeners) as previously described (Bloom et al. 2007). Briefly, gas chromatography with electron capture detection (GC-ECD) was used to analyze serum samples in batches of 10 with four additional quality control (QC) samples. These QC samples comprised one reagent blank, one serum blank, one sample containing sheep serum and 15 calibration standards at known values, and one participant’s duplicate sample.

Children’s serum was analyzed for the quantification of 209 PCB congeners by high-resolution gas chromatography/high-resolution mass spectrometry (HRGC/HRMS) as performed by AXYS Analytical Services, Ltd. (Sidney, British Columbia, Canada), using U.S. Environmental Protection Agency (EPA) method 1668A (U.S. EPA 2008). Briefly, serum is spiked with ^13^C-labeled surrogate standards before solvent extraction and instrumental analysis. Extracts are subject to chromatographic cleanup procedures. The quality assurance component included three controls: one reagent blank, one spiked reference blank, and one participant’s duplicate sample. This laboratory met the World Health Organization criteria for analysis of PCBs in human milk and plasma (Stephens et al. 1992).

All laboratory values were corrected only for recovery to minimize measurement error and to avoid potential biases associated with substitution of values below the limit of detection or the automatic adjustment for lipids (Schisterman et al. 2006; Whitcomb and Schisterman 2008). Therefore, all machine-read values were included in the analyses without substitution for values below the limit of detection, which differed by congener and batch.

### Statistical analysis

PCB congeners were expressed in nanograms per gram of serum wet weight comparable with parts per billion. In an attempt to assess PCB mixtures in the context of our limited cohort size, we used an *a priori* classification scheme for grouping congeners by purported biologic activity (Cooke et al. 2001), given the absence of universally accepted groupings of congeners by biologic activity. We first summed all measured congeners into a simple total and then categorized them into estrogenic, antiestrogenic, and other congener groupings. The sum of estrogenic congeners included congeners 4_10, 5_8, 15_17, 18, 31, 44, 47, 48, 52, 70, 99, 101, 136, 153, and 188, whereas the sum of antiestrogenic congeners included congeners 77_110, 105, 114, 126, 156_171, and 169 (underscores indicate dielution). The other congener grouping comprised congeners 6, 7_9, 16_32, 19, 22, 24_27, 25, 28, 33, 40, 42, 45, 50, 55, 59, 60, 64, 66_95, 74, 81_87, 82, 94, 97, 118, 128, 129, 132, 134, 135, 138, 141, 147, 149, 151, 157_200, 163, 167, 170, 172, 174, 176, 177, 179, 180, 181, 183, 185, 187, 189, 190, 194, 195, 196_203, 205, and 206. PCB concentrations were log transformed to meet the normality assumptions for our statistical models and also were categorized into tertiles.

We quantified total serum lipids (TL) using enzymatic methods as the function of total cholesterol (TC), free cholesterol (FC), triglycerides (TG), and phospholipids (PL) using the formula TL = 1.677 (TC – FC) + FC + TG + PL (Phillips et al. 1989). We did not automatically adjust serum PCB concentrations for lipids, given the causal assumption implicit in this approach (Schisterman et al. 2005). Lipid adjustment implies that lipids are causally related to birth weight and exposure. However, we were unaware of any empirical evidence to support this strong assumption necessitating the use of unadjusted concentrations in statistical models. Moreover, recent evidence suggests that such approaches introduce a form of bias (Schisterman et al. 2005). Given the remaining controversy regarding how best to model serum lipids for nonfasting samples, we assessed maternal serum lipids as a covariate in the statistical models (although no relation was observed supporting our *a priori* decision). For the analyses comparing maternal and children’s serum concentrations, we restricted the data to the 76 congeners common to both matrices and lipid-adjusted all concentrations, given that we were comparing different media with varying lipid concentrations rather than health outcomes per se.

After inspection of the graphical displays of continuous exposure data and birth weight, we used various descriptive statistics to fully assess the quality of the data and to obtain measures of central tendency and dispersion. We used ordinary least squares (OLS) linear regression to identify determinants of birth weight (grams) and gestation (days), including the relation between maternal PCB concentrations and birth weight. We conducted separate analyses for PCB concentrations as both continuous natural log–transformed (nanograms per gram serum) and categorized (tertiles) exposures. Preconception and prenatal PCBs were modeled separately for each PCB grouping (i.e., total, estrogenic, antiestrogenic, and other congeners). Postnatal PCB concentrations were not analyzed in relation to birth weight, given that they were quantified at a median of 6 weeks after delivery. Hence, we could not support the temporal ordering between PCB exposure and outcome.

We inspected OLS regression residuals to verify that a linear model was appropriate. Given our limited cohort size, we adjusted only for covariates relevant for the exposures and outcome (i.e., maternal preconception cigarette smoking, height, serum lipids, and infant’s sex) and retained those that altered the exposure’s coefficient by > 15%. We modeled cigarette smoking as a dichotomous variable and also in categories based on the observed distribution (0, 1–5, and > 5 cigarettes per day) and found little difference in the estimated values of the parameter associated with the exposure. Thus, we chose the dichotomous categorization to support a more parsimonious model in light of our limited sample size. *A priori*, we decided not to adjust for gestational age when estimating PCB effects on birth weight because gestational age is in the causal pathway and not a confounder per se (Weinberg 1993). Such an adjustment has been empirically demonstrated to introduce bias (Schisterman et al. 2009). We explored a potential interaction between infant sex and PCB concentrations, in response to earlier reports suggesting male susceptibility for decrements in birth weight (Sonneborn et al. 2008), by including an interaction term in the adjusted models and assessing the *p*-value obtained by the Wald chi-square test. Lastly, we assessed maternal serum PCB concentrations before, during, and after pregnancy in relation to children’s serum concentrations at 2 years of age along with duration of breast-feeding to estimate the variation explained by each. The percentage of explained variation for each sensitive window and breast-feeding duration in relation to children’s serum concentration was calculated by dividing each model coefficient’s type III sums of squares by the total model type III sum of squares. For example, the percentage of variation explained by maternal preconception concentrations was estimated as the preconception PCB coefficient sum of squares (0.15) divided by the total model sum of squares (0.679) = 22%. Children who were not breast-fed were assigned a breast-feeding duration of 0 days.

## Results

The cohort comprised white married women who were largely college educated (67%) and nonsmokers at the time of cohort commencement (72%) (Table 1). Mean ± SD birth weight and gestational age for the cohort’s infants were 3,500 ± 580 g and 258 ± 14 days, respectively. To this end, 6.0% of infants were classified as having a low birth weight (< 2,500 g), and 9.1% of infants were classified as preterm (gestational age < 245 days from the date of conception). Other than gestational age, no other covariates were significantly associated with birth weight, although patterns for female sex and cigarette smoking were in the expected direction: Female infants and those born to mothers who reported smoking before conception had lower birth weights than their respective counterparts.

Mean total PCB concentrations were higher at baseline (preconception) than during the prenatal period (i.e., 5.6 and 4.7 ng/g serum, respectively) (Table 2). The relation between preconception PCB concentrations and birth weight varied by PCB grouping, with antiestrogenic PCBs conferring an approximately 471-g reduction in birth weight for women in the highest versus lowest tertile of exposure when adjusting for study covariates (*p* = 0.048), but no such decrement for estrogenic PCBs (Table 3). This pattern remained when assessing prenatal concentrations in tertiles, although the results did not achieve significance. When we assessed total PCBs or PCB groupings as continuous log-transformed exposures, we found no significant associations; however, all β-coefficients were negative (total PCBs: β = −400, *p* = 0.29; antiestrogenic PCBs: β = −100, *p* = 0.40; estrogenic PCBs: β = −700, *p* = 0.16; other PCBs: β = −300, *p* = 0.39).

In an attempt to delineate which antiestrogenic PCB congeners may be driving the reduction in birth weight, we analyzed each congener (congeners 77_110, 105, 114, 126, 156_171, 169) in relation to birth weight. We observed no significant associations between specific congeners and birth weight, although most associations were negative (data not shown). However, preconception PCB-105 concentration was associated with the largest individual reduction in birth weight when comparing women in the highest versus lowest tertile (β = −370 g, *p* = 0.075) or in its continuous form (β = −380, *p* = 0.054, for unit change in nanograms per gram PCB on the natural log scale). We explored a possible interaction for PCBs (continuous and by grouping) and infant sex but observed no significant effects in either the unadjusted or adjusted models. In a secondary analysis, we examined PCB concentrations in relation to gestational length in days from conception without observing an association irrespective of PCB groupings (third vs. first tertile: total preconception PCBs, unadjusted β = 0.87, *p* = 0.84; total prenatal PCBs, unadjusted β = 0.5, *p* = 0.90). Results were largely unchanged after adjustment for maternal age and cigarette smoking.

Last, we explored maternal serum concentrations at sensitive windows and duration of breast-feeding in relation to the children’s serum concentrations at 2 years of age to estimate the correlation of maternal–child serum concentrations, as well as the variation explained in children’s concentrations by maternal concentrations at varying sensitive windows. Table 4 presents four models, starting with preconception and adding each successive sensitive window (prenatal and postnatal), with model 4 including all three sensitive windows plus breast-feeding duration. Maternal preconception PCB concentrations and breast-feeding duration explained 15–22% and 32%, respectively, of children’s concentrations at age 2 years. Importantly, 56% of children’s PCB concentrations were explained by maternal serum concentrations during the preconception, prenatal, and postnatal sensitive windows and duration of breast-feeding. The correlation coefficients for children’s PCB concentrations and maternal PCB concentrations were 0.47 (preconception), 0.16 (prenatal), and 0.33 (postnatal). We observed a correlation of 0.53 for duration of breast-feeding and children’s PCB concentrations.

## Discussion

In this population-based prospective cohort study with preconception enrollment of women, three important findings emerge that add to the evolving body of evidence suggesting possible developmental toxicity of PCBs, particularly those purported to have antiestrogenic activity. First, we observed a large reduction (~ 470 g) in birth weight for antiestrogenic PCBs during the preconception window, a time period unaffected by physiologic changes associated with pregnancy, including those that precede a woman’s recognition of pregnancy. The magnitude of effect is considerably more than that reported for cigarette smoking (~ 55–189 g reduction) (Cliver et al. 1995), underscoring its importance for public health. Furthermore, we observed no significant reduction in gestational age in relation to PCB groupings or concentrations despite having a valid estimate of conception. This finding suggests that the reduction in birth weight is not mediated through gestation, but instead through a pathway directly affecting fetal growth.

A second key finding is the importance of congener groupings when assessing PCBs and human development as measured by birth weight. Estrogenic PCBs were not negatively associated with birth weight in our cohort and may explain, in part, the equivocal findings across studies, particularly those that do not differentiate their exposures by purported biologic activity or that rely on simple sums of PCBs or select congeners (Ribas-Fito et al. 2002; Wolff et al. 2007). Last, our findings provide empirical support that maternal exposures affect children’s exposures regardless of breast-feeding. As previously stated, duration of breast-feeding explained a considerable percentage, but not all, of children’s serum concentrations.

Despite increasing recognition about critical or sensitive windows of human development (Chapin et al. 2004; Johnson et al. 2006; Louis et al. 2008), we are unaware of other prospective cohort studies enrolling women/couples before conception for the quantification of PCBs. Thus, it is difficult to fully interpret our data relative to others’ work. Prior investigators have assessed maternal PCB concentrations in relation to birth weight, but typically have relied on blood collected at varying times during pregnancy or at delivery (Hertz-Picciotto et al. 2005; Karmaus and Zhu 2004; Sonneborn et al. 2008) or on cord blood or breast milk (Patandin et al. 1998; Tan et al. 2009). Collectively, past research suggests a decrement of 100–300 g in birth weight for a subset of quantified PCBs in serum (Hertz-Picciotto et al. 2005). Our effect is larger for the antiestrogenic PCBs when quantified before conception, although the effect remains apparent during early pregnancy. A recent study using data from this cohort reported a decline in serum concentration from preconception to postnatal period (Bloom et al. 2007), suggesting the importance of timing biospecimen collection for assessing developmental toxicity. However, corroboration of these findings is needed to better inform investigators regarding when (and how often) to collect maternal biospecimens when assessing developmental toxicity.

We are unaware of research efforts that have quantified PCBs by purported biologic activity or that have focused on antiestrogenic PCBs, more specifically, in relation to birth weight, possibly given the limited number of congeners selected in prior research or the reliance on simple sums of select congeners (Hertz-Picciotto et al. 2005; Patandin et al. 1998; Sonneborn et al. 2008; Tan et al. 2009). Regardless, the available evidence suggests an adverse relation between maternal PCBs and birth weight (Halldorsson et al. 2008; Hertz-Picciotto et al. 2005; Karmaus and Zhu 2004; Patandin et al. 1998; Sonneborn et al. 2008). Previous studies reporting reductions in birth weight have generally reported PCB concentrations relatively similar to ours, although often with fewer measured congeners. For example, median total PCB concentrations have ranged from 1.11 μg/L plasma (Halldorsson et al. 2008) or 2.0 μg/L plasma (Patandin et al. 1998) to 5–8 μg/L wet weight serum (Karmaus and Zhu 2004; Sonneborn et al. 2008). Our pregnancy cohort had a range of PCB concentrations, although a median total prenatal PCB concentration of 4.5 (range, 3.8–5.5) ng/g serum, which is comparable with the value reported by the U.S. Collaborative Perinatal Project [4.4 (3.3–6.1) μg/L serum (Longnecker et al. 1999)], assuming nanograms per gram are comparable with micrograms per liter, with both representing parts per billion.

Estrogenic PCB congeners may disrupt endocrine activity by competitive binding with estrogenic receptors or by disrupting enzymes critical to estrogenic processes, whereas antiestrogenic PCB congeners may inhibit the effects of endogenous estrogens (Cooke et al. 2001; Kester et al. 2000). Furthermore, estrogens in pregnancy are associated with increased birth weight (Kaijser et al. 2000; Nagata et al. 2006), so PCB congeners with purported antiestrogenic bioactivity may disrupt estrogenic pathways, potentially leading to a decrease in birth weight. In addition, antiestrogenic congeners, such as PCB-105, have been implicated in thyroid-mediated effects due to high affinity for thyroid hormone–binding proteins, leading to disruptions in thyroid hormone signaling (Crofton et al. 2005), which may also disrupt growth and development (Sahu et al. 2009).

The absence of prior research with longitudinal measurement of PCBs during the preconception sensitive window coupled with quantification in children limits our ability to fully interpret our estimated explained variation in children’s serum concentrations relative to mothers’ concentrations during sensitive windows of development. The plausibility of the preconception window has been noted for other environmental exposures, such as the Dutch famine. Specifically, offspring whose mothers were affected by undernutrition around the time of conception were more likely to have shorter gestations than were offspring whose mothers were not affected (38.6 vs. 39.0 gestational weeks, respectively; *p* < 0.0001) and also were more likely to have coronary heart disease and obesity as adults, underscoring the importance of the pre- and periconception window for human development (Painter et al. 2005; Rayco-Solon et al. 2005). Previous authors have assessed breast-feeding in relation to children’s exposures and generally report higher levels among breast-fed children (Jacobson et al. 1989; Lanting et al. 1998). We observed a similar finding, with breast-fed children having a higher mean ± SD PCB concentration at 2 years of age compared with formula-fed children (1.83 ± 1.6 ng/g and 0.41 ± 0.2 ng/g, respectively). Combined, these data support breast-feeding as a postnatal route of PCB exposure for nursing infants.

To our knowledge, only one prior study has modeled infant PCB concentrations as a function of maternal PCB concentrations at delivery or postnatally. Ayotte et al. (2003) analyzed maternal plasma concentrations of PCB-153 approximately at delivery and breast-feeding duration in relation to infant concentrations at age 6 months and reported that the model explained 66% of variation in infant PCB-153 plasma concentration. In our cohort, maternal preconception PCB concentrations explained the highest variation in children’s concentrations (15%), followed by prenatal (2%) and postnatal (< 0.1%) PCB concentrations. Breast-feeding duration increased the total explained variation to 50%, suggesting that maternal exposures account for half of children’s exposure at 2 years of age. Our estimate is lower than that reported by Ayotte et al. (2003) and may reflect the difference in age when children’s concentrations were quantified or the choice of PCB congener(s) analyzed. Because children in our cohort were approximately 24 months of age when PCB concentrations were quantified, other environmental sources may contribute to their concentrations, particularly diet or play. In addition, we recognize the imprecision in PCB concentrations particularly at lower limits given our use of GC-ECD for quantification of maternal serum, although we were able to use the HRGC/HRMS for quantifying children’s serum.

Several important limitations warrant cautious interpretation of the findings until larger prospective studies with longitudinal measurement of exposures during sensitive windows are available. Despite its limited size, the cohort was sufficiently powered to detect large differences in birth weight, which are at least twice the effect typically observed for cigarette smoking. However, we had limited power to assess other potential covariates or even a possible interaction with sex, which is concerning given that PCBs were previously associated with reduced secondary sex ratios or the number of male and female births in this cohort (Taylor et al. 2007). In another study, the association between maternal PCB concentrations and infant birth weight differed between male and female infants (Sonneborn et al. 2008). We were underpowered to assess the relation between daily rate of change in PCBs and birth weight, although we did assess changes in women’s tertile rankings across sensitive windows. The percentages of women remaining in the same tertile for preconception and prenatal PCB concentrations were highest for antiestrogenic PCBs (58%), followed by other (47%), total (44%), and estrogenic (37%) PCBs. We did not have any other measures of birth size, such as length or head or abdominal circumferences, which prevented us from assessing the proportionality of infants. We did have an excellent estimate of conception, given the longitudinal capture of menstruation, sexual intercourse, and hCG-confirmed pregnancy. Other study limitations include maternal self-reported information on birth weight and the potential for residual confounding by cigarette smoking during pregnancy. However, we expect that accuracy of maternal reporting of birth weight is not related to PCB concentrations and therefore may introduce error but not bias per se. We recognize the potential for selection bias but did not have exposure data on women not participating to empirically assess its potential effect. Because women were unaware of their PCB concentrations, we assume that participation was not systematically associated with PCB exposure.

As the literature continues to evolve in support of an association between PCB exposures during sensitive windows of fetal growth and development as measured by birth weight, it is essential that mechanistic research be conducted to better inform how best to protect vulnerable subgroups of the population. Determining whether and how such mechanisms disproportionately affect males (or females) remains to be established.

## Conclusions

Our prospective cohort study with longitudinal measurement of maternal PCBs underscores the preconception interval as a sensitive window for human development, and the importance of quantifying a mixture of PCBs by purported biologic activity. Our findings underscore that preconception maternal exposures at environmentally relevant concentrations are adversely associated with fetal growth as approximated by birth weight and serve as a route of exposure for children along with breast-feeding.

## Figures and Tables

**Table 1:** Maternal characteristics and associations with birth weight (*n* = 55).

Characteristic	Value	β	SE
Education			
Less than college	18 (33)	Reference	
College graduate or higher	37 (67)	120.4	183.4
Gravidity			
Nulligravida	8 (15)	Reference	
Multigravida	47 (85)	140.6	215.9
Parity			
Nulliparous	10 (18)	Reference	
Multiparous	45 (82)	173.8	225.8
Preconception cigarette use			
No	38 (72)	Reference	
Yes	15 (28)	−263.8	193.5
Infant sex			
Male	29 (47)	Reference	
Female	26 (53)	−250.5	175.4
Maternal age (years)	30 ± 2.3	6.9	38.3
Preconception BMI (kg/m^2^)	24 ± 4.8	5.7	16.7
Maternal height (inches)	65 ± 2.8	17.2	29.4
Preconception serum lipids (mg/dL)	4.2 ± 1.4	39.1	69.9
Prenatal serum lipids (mg/dL)	4.2 ± 1.5	94.9	56.8
Gestational age (days)	258 ± 14	6.3*	4.8

BMI, body mass index. Values are no. (%) or mean ± SD. Missing data were uniformly excluded from analyses. β-Values, SEs, and *p*-values were estimated using linear regression of characteristics on birth weight (grams).

* *p* < 0.001.

**Table 2:** Maternal serum PCB concentrations during sensitive windows.

	Preconception^a^			Prenatal^b^		
Congener grouping (tertiles ng/g serum)	Mean	Median	IQR	Mean	Median	IQR
Total PCBs	5.6	5.3	5.0–6.2	4.7	4.5	3.8–5.5
Low	4.5	4.6	4.1–5.0	3.5	3.6	3.3–3.8
Medium	5.4	5.3	5.1–5.4	4.5	4.5	4.2–4.8
High	6.9	6.3	6.2–7.0	5.9	5.8	5.5–6.2

Antiestrogenic PCBs	0.22	0.20	0.15–0.23	0.13	0.11	0.09–0.15
Low	0.13	0.15	0.13–0.15	0.08	0.08	0.07–0.09
Medium	0.20	0.20	0.19–0.20	0.11	0.11	0.11–0.11
High	0.32	0.28	0.23–0.33	0.20	0.17	0.15–0.21

Estrogenic PCBs	2.3	2.2	2.1–2.4	1.9	1.9	1.6–2.2
Low	1.9	1.9	1.7–2.1	1.5	1.4	1.4–1.6
Medium	2.2	2.2	2.1–2.3	1.9	1.9	1.8–2.0
High	2.7	2.7	2.5–2.7	2.4	2.4	2.2–2.5

Other PCBs	3.1	2.9	2.6–3.4	2.6	2.6	2.1–3.1
Low	2.4	2.5	2.3–2.6	2.0	1.9	1.8–2.1
Medium	2.9	2.9	2.8–3.0	2.5	2.6	2.4–2.7
High	4.0	3.6	3.4–3.9	3.4	3.3	3.2–3.4

a *n* = 50.

b *n* = 52.

**Table 3:** Maternal serum PCB concentrations and birth weight [β (95% CI)].

	Preconception		Prenatal	
PCB concentration (tertiles ng/g serum)	Unadjusted	Adjusted^a^	Unadjusted	Adjusted^a^
Total PCBs				
Medium	−309.3 (−701.9 to 83.4)	−306.8 (−696.9 to 83.3)	−105.0 (−497.0 to 286.9)	−119.1 (−512.0 to 273.9)
High	−237.6 (−630.2 to 155.1)	−205.5 (−618.1 to 207.0)	−120.8 (−505.5 to 263.9)	−140.9 (−543.1 to 261.3)

Antiestrogenic PCBs				
Medium	2.1 (−375.9 to 386.0)	−11.0 (−391.1 to 369.1)	89.3 (−290.0 to 468.7)	58.9 (−324.1 to 441.9)
High	−429.3 (−807.2 to −51.4)*	−470.8 (−890.2 to −51.3)*	−248.9 (−628.2 to 130.5)	−260.5 (−667.4 to 146.5)

Estrogenic PCBs				
Medium	167.6 (−225.7 to 560.9)	281.6 (−108.8 to 672.1)	65.3 (−320.8 to 451.5)	118.3 (−276.3 to 513.0)
High	15.0 (−399.7 to 429.6)	34.7 (−373.0 to 442.4)	21.4 (−372.0 to 414.8)	3.2 (−420.3 to 426.6)

Other PCBs				
Medium	−150.2 (−555.1 to 254.7)	−125.4 (−529.1 to 278.4)	1.2 (−385.8 to 388.3)	−51.1 (−453.1 to 350.8)
High	−255.5 (−645.3 to 134.2)	−235.6 (−636.1 to 165.0)	−220.0 (−599.9 to 159.9)	−200.0 (−591.2 to 191.1)

CI, confidence interval. Low tertiles were used as the referent category in linear regression models. The valid sample number is 50 for the preconception models and 52 for the prenatal models.

a Adjusted for maternal height, maternal preconception smoking (yes/no), and infant’s sex.

* *p* < 0.05.

**Table 4:** Variation in children’s serum PCB concentrations at 2 years of age explained by maternal PCB concentrations during sensitive windows (*n* = 19).

Maternal PCB concentration (ng/g lipid)					Model *R*^2^
Model 1: preconception PCBs					0.22
Explained variation	22%				

Model 2: preconception PCBs + prenatal PCBs					0.26
Explained variation	25%	6%			

Model 3: preconception PCBs + prenatal PCBs + postnatal PCBs					0.28
Explained variation	20%	8%	2%		

Model 4: preconception PCBs + prenatal PCBs + postnatal PCBs + breast-feeding duration					0.56
Explained variation	15%	2%	< 1%	32%	
